# Effects of the COVID-19 Infection on Women's Menstrual Cycle: A Retrospective Study at Latifa Hospital, Dubai, United Arab Emirates

**DOI:** 10.7759/cureus.51391

**Published:** 2023-12-31

**Authors:** Nighat Aftab, Asma Fahad, Safia Al Hammadi, Muna Tahlak, Faiza Badawi, Israa Al Mulai, Saima Faraz, Sofia Malik, Parveen Mohammed, Sadia Maqbool, Zoiya Fatima, Esraa Osman

**Affiliations:** 1 Obstetrics and Gynaecology, Latifa Women and Children Hospital, Dubai Academic Health Corporation, Dubai, ARE; 2 Obstetrics and Gynaecology, Dubai Academic Health Corporation, Dubai, ARE; 3 Obstetrics and Gynaecology, Dubai Health Authority, Dubai, ARE; 4 Obstetrics and Gynaecology, Latifa Women and Children Hospital, Dubai, ARE

**Keywords:** covid-19, post-menopausal bleeding, intermenstrual bleeding, dysmenorrhea, menorrhagia

## Abstract

Background

The COVID-19 pandemic has affected all of us in one way or another. The menstrual cycle is a reflection of the female reproductive system, and it is influenced by various factors including stress and infections. Although there is little information available about how the COVID-19 pandemic has affected women's reproductive health, it has had a significant impact on women.

Objective

The main objective of our study is to identify if there are any menstrual disturbances following COVID-19 infection in women.

Study design

Our study is a retrospective study wherein 700 women recovering from COVID-19 infection were asked about any new menstrual disturbances after the infection. We collected the data using questionnaires and analyzed the data using Statistical Product and Service Solutions (SPSS, version 26) (IBM SPSS Statistics for Windows, Armonk, NY).

Results

Our study showed no dysmenorrhea in 90% of the participants, 81% reported no change in menstrual flow rate, a vast majority (93%) denied experiencing amenorrhea, only 4% reported a new onset of intermenstrual bleeding, and 1% reported postmenopausal bleeding.

Conclusion

There was no significant change in the participants’ menstrual cycle following COVID-19 infection.

## Introduction

COVID-19 hit us in early 2020, started as a fatal pandemic, and has now become a part of our everyday lives. Although the virus has now been investigated extensively, some of its effects still elude us. There is a paucity of data regarding the effect of COVID-19 infection on the menstrual cycle.

The menstrual cycle is a reflection of the female reproductive system, and it is influenced by various factors including stress and infections, and there is little information available on how the COVID-19 pandemic has affected women's reproductive health, and the long-term health implications of this have yet to be determined [1]. COVID-19 survivors are shown to have moderate to severe symptoms of PTSD, anxiety, or depression [2]. It has been shown that COVID-19 infection causes menstrual changes without long-term consequences [3]. 

## Materials and methods

Aims

Our research attempts to understand how the COVID-19 infection affects the menstrual cycle in women.

Design

This is a cross-sectional study.

Objectives

The objective of this study was to identify if women have noted any change in their menstrual pattern after COVID-19 infection to assess COVID-19's impact on the menstrual cycle. As COVID-19 remains a largely unknown entity with widespread and mostly unknown effects on many organ systems, this study was designed to evaluate its effects on the female reproductive system.

Research design and methods

Data was collected from reproductive-aged women (ages 18-55) who have recovered from COVID-19 infection using a survey form. Our electronic record system was used to identify women between the ages of 18-55 who were positive for COVID-19 during 2020-2021, and 1000 patients were selected from this list.

Study design and methodology

Healthcare professionals contacted the individuals and filled out the questionnaire forms after obtaining verbal consent regarding COVID-19 infections and any effects on menstrual cycles. The questionnaires included close-ended questions about age, ethnicity, history of infection, severity, and treatment and were followed by questions regarding any changes noted by patients in their menstrual cycle including flow, regularity, dysmenorrhea, intermenstrual bleeding, or postmenopausal bleeding in cases of postmenopausal pain. The questionnaire is added to the Appendix. Data was analyzed using Statistical Product and Service Solutions (SPSS) (version 26; IBM SPSS Statistics for Windows, Armonk, NY).

Research population and sample size calculation

We aimed to collect data from 1,000 participants between the ages of 18 and 55. Through our electronic record system, we obtained the names of patients with COVID-19 infections. Healthcare professionals contacted these individuals and provided them with questionnaire forms after obtaining verbal consent.

Limitations

We aimed to collect data from 1,000 patients; however, 295 patients either no longer resided in the UAE or refused to participate in the study.

Ethical considerations

The Dubai Scientific Research and Ethical Committee approved this study.

## Results

This study's objective was to assess COVID-19's impact on the female menstrual cycle. Healthcare professionals contacted the individuals and filled out the questionnaire forms after obtaining verbal consent regarding COVID-19 infections and any effects on menstrual cycles. A total of 705 participants took part in the survey. This chapter presents the findings of the survey, including demographic information, COVID-19 symptoms experienced by the participants, treatment received, and impact on the menstrual cycle. The analysis aims to provide insights into the relationship between COVID-19 and the female reproductive system.

Demographic profile

**Figure 1:**
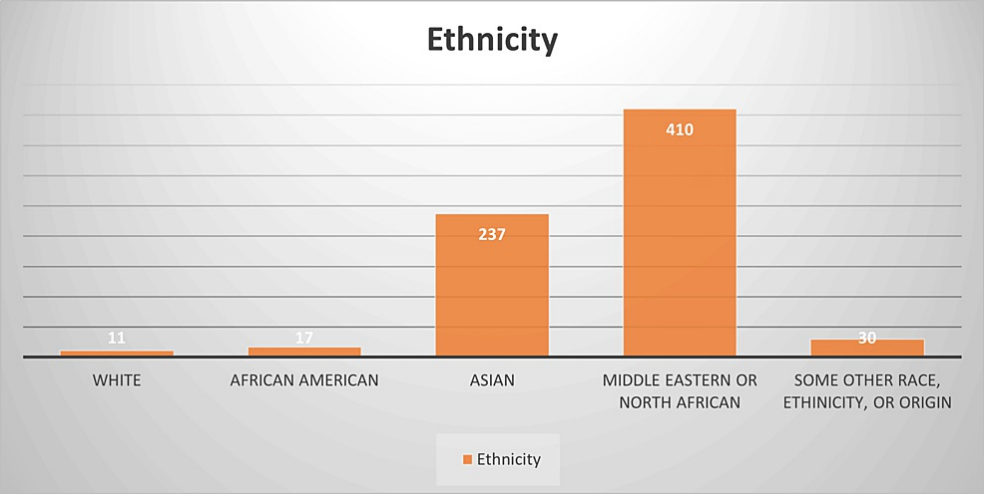
Ethnicity

The survey participants consisted of 705 women. The majority of respondents fell in the age range of 30-35 years (31%). Regarding menopause status, 97% of the participants (681 individuals) reported not being menopausal. Middle Eastern or North African women accounted for 58% (410 persons) of the total respondents (see Figure 1).

Furthermore, 74% of the participants stated that they had no chronic conditions, whereas 27% reported having chronic conditions, including obesity, diabetes mellitus, pulmonary disease, dyslipidemia, cardiac disease, anxiety/depression, or cancer.

COVID-19 symptoms

**Figure 2:**
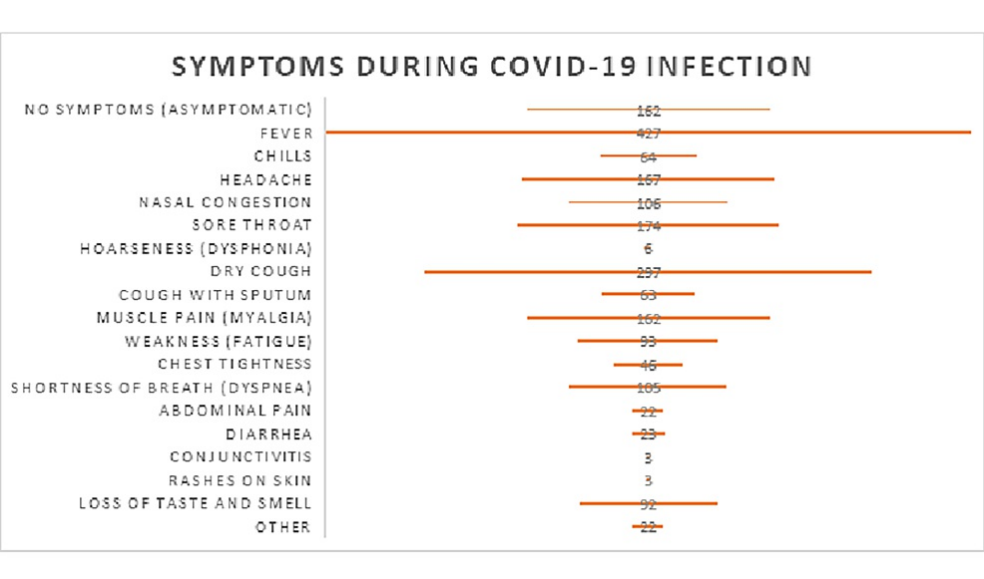
Symptoms during COVID-19 infection

When asked about symptoms experienced during COVID-19 infection, the responses varied. Out of the 705 participants, 162 individuals reported no symptoms (asymptomatic). The most commonly reported symptoms were fever (427 persons), headache (167 persons), sore throat (174 persons), nasal congestion (106 persons), and dry cough (297 persons). Other less frequently reported symptoms included chills, hoarseness, cough with sputum, muscle pain, weakness, chest tightness, shortness of breath, abdominal pain, diarrhea, conjunctivitis, rashes on the skin, and loss of taste and smell (Figure 2).

Treatment and medication

Of the participants, 57% were admitted to the hospital, 28% received treatment at home, 13% were treated at home under doctor consultation, and 1% did not answer this question. Among those treated at home or under doctor consultation, 59% did not take any medication, and 41% reported taking medicines. For those admitted to the hospital, 73% stayed less than five days and 27% stayed 5-10 days. Oxygen treatment was received by 86 individuals, high-flow nasal cannula by 33 individuals, mechanical ventilation by 11 individuals, and other medications by 156 individuals. Regarding COVID-specific medication, 58% did not receive any, whereas 42% received specific medications.

Impact on menstrual cycle

In terms of the menstrual cycle, the survey asked specific questions regarding dysmenorrhea, menstrual flow rate, amenorrhea, intermenstrual bleeding, and postmenopausal bleeding. Regarding new-onset dysmenorrhea, 90% of the participants reported not experiencing painful periods after having COVID-19, whereas only 10% reported experiencing pain. Similarly, 81% reported no change in menstrual flow rate, whereas 19% reported a change. A vast majority (93%) denied experiencing amenorrhea, whereas only 7% reported it. Only 4% reported a new onset of intermenstrual bleeding, whereas 96% did not. Only 3% of women were postmenopausal, and 1% reported postmenopausal bleeding.

## Discussion

As of today, there have been 767,972,961 confirmed cases of COVID-19, including 6,950,655 deaths as reported to the WHO [4]. Where now we have a significant body of research on COVID-19 and its diagnoses, management, and treatment, there has been little research on its effects on the menstrual cycle. There have been studies that have focused on the impact of COVID-19 vaccination on the female reproductive cycle, but only a handful of studies on COVID-19 infection and its effect on menstrual irregularities.

There has been some speculation on menstrual cycle irregularity after COVID-19 infection. Unfortunately, menstruation is an understudied research topic, and there is a literature gap in menstrual changes after COVID-19 infection [5]. Little is known about the influence of COVID-19 on the menstrual cycle. A study by Khan et al. showed a positive relationship between the severity of infection and menstrual disturbances; however, the study was limited due to its small sample size [6].

Multiple respiratory viruses elicit different immune responses between the males and females. Females have been shown to mount a greater cytokine storm after influenza infection, and, thus, take longer to recover from the infection [7]. These variations might also influence the course of COVID-19 infection. The association of the immune system on the female sex hormones is not well-understood [8].

The hypothalamic-pituitary-adrenal (HPA) axis is the first neuroendocrine axis to be altered due to viral infections and stressful situations to the body [9]. Abnormal uterine bleeding may result from endometrial immune cells dysregulation [10]. The interaction between HPA and hypothalamo-pituitary gonadal (HPG) axis makes it possible for the immune response-mediated stress due to COVID-19 to cause transient changes in the menstrual cycle [8]. Case reports have linked COVID-19 vaccine to pituitary disorders. A study by Taieb et al. reviewed eight case reports of pituitary disorder that developed after the vaccine. Regarding the types of pituitary disorder, five were hypophysitis (variable clinical aspects ranging from pituitary lesion to pituitary stalk thickness), and three were pituitary apoplexy [11].

**Figure 3:**
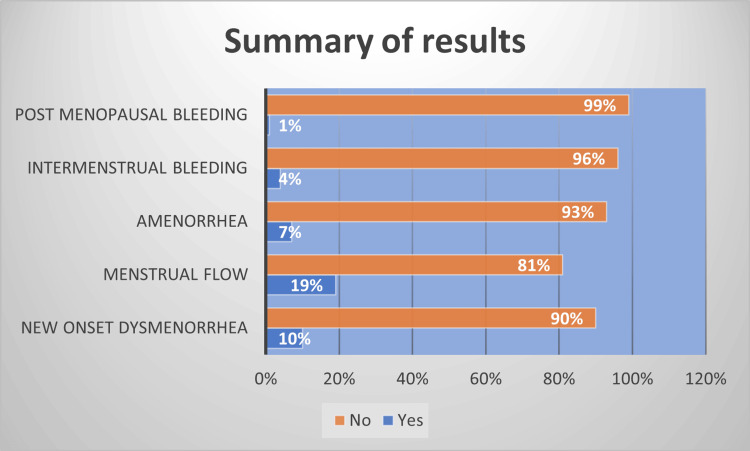
Summary of results

We focused on common menstrual problems, such as dysmenorrhea, change in flow, new-onset amenorrhea, intermenstrual bleeding, and postmenopausal bleeding in postmenopausal women. A summary of the results is shown in Figure 3.

**Figure 4:**
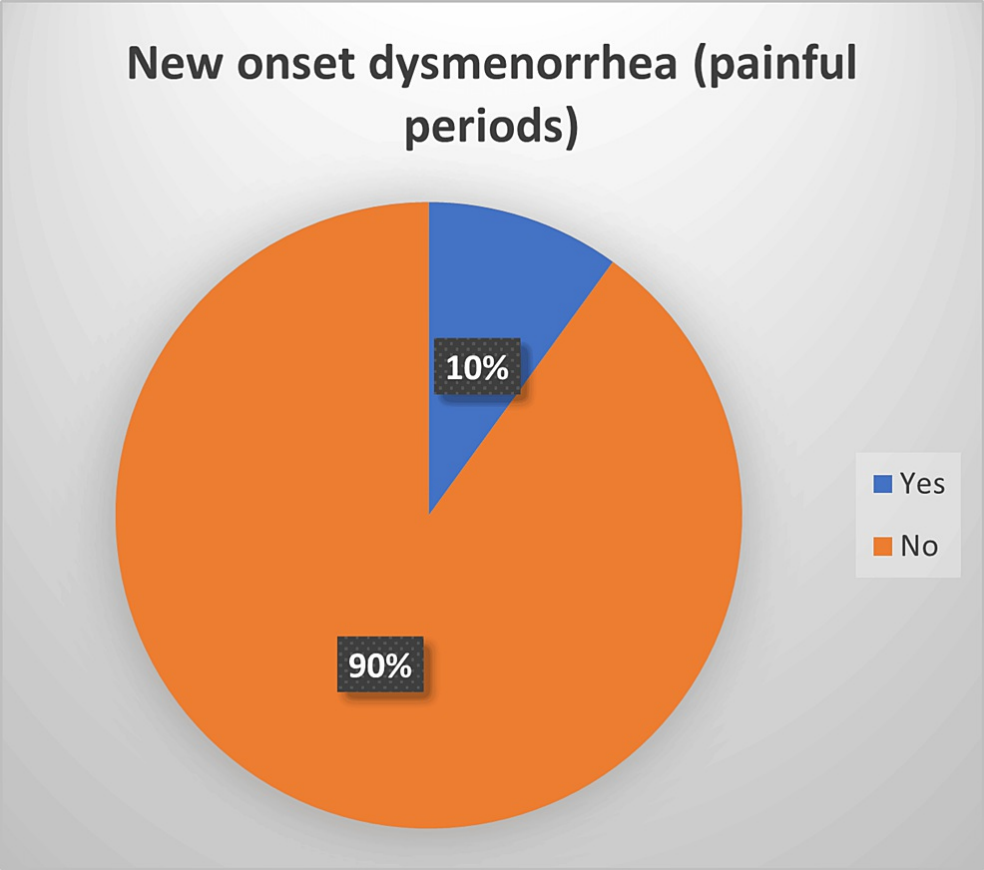
New-onset dysmenorrhea

In our study, 90% of the patients denied having new-onset dysmenorrhea, with 10% reporting this new symptom occurring after COVID-19 infection (see Figure 4). This is consistent with the study by Chourasia et al., which noted a significant increase in dysmenorrhea during COVID-19 infection, but the levels returned to normal after the infection [12].

**Figure 5:**
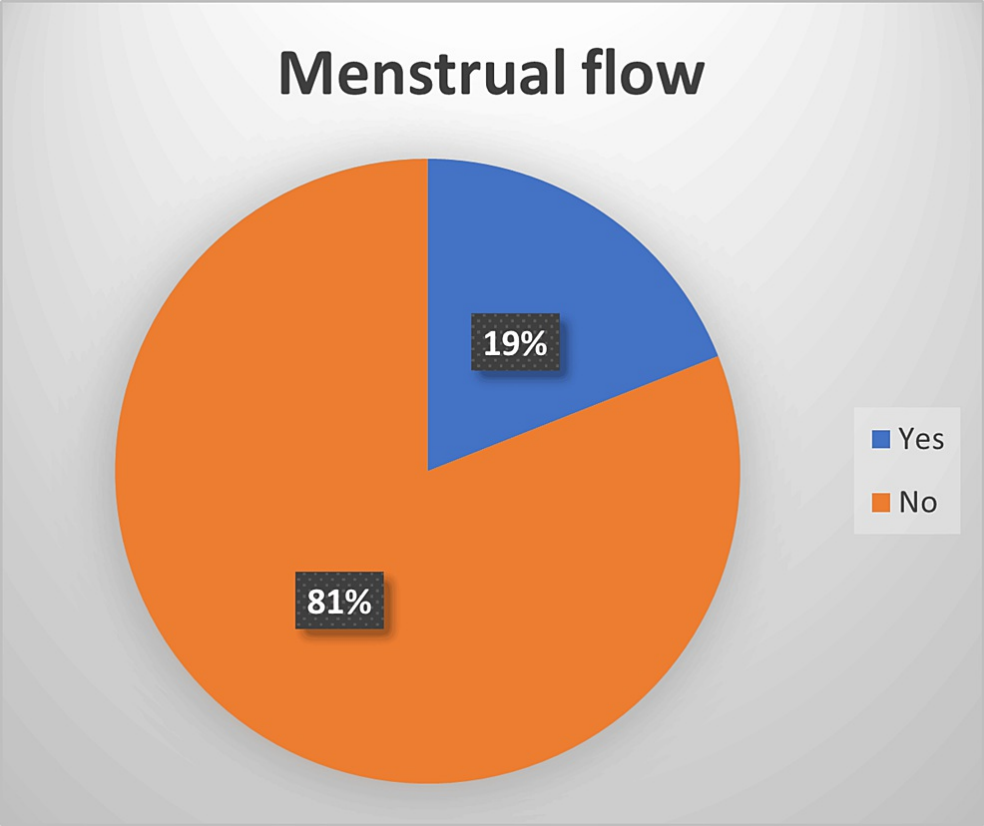
Menstrual flow

Our study looked into the effect of menstrual flow noted after infection, in which 19% of patients reported an impact on flow (see Figure 5). A study by Li et al. showed that, while 20% of women had a considerable reduction in menstrual volume, with no discernible difference between patients who were mildly or severely ill, a lesser percentage of patients experienced menorrhagia or shortening of their menstrual cycle [13].

**Figure 6:**
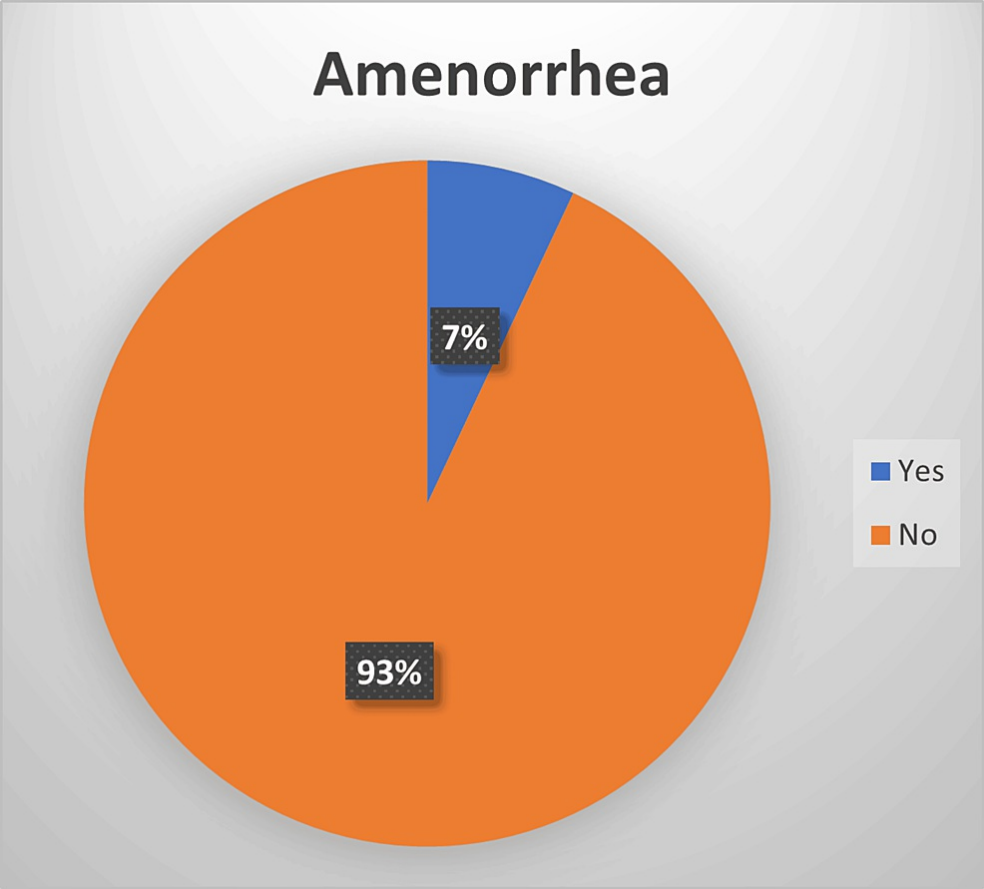
Amenorrhea

Only 7% of our patients experienced amenorrhea after infection (see Figure 6), which is concurrent with the findings in the study done by Ding et al., but it showed that women who suffered from more severe COVID-19 infections had higher levels of amenorrhea, menorrhagia, abnormal uterine bleeding, and dysmenorrhea in contract to less severe cases, but the differences were not significant [14].

**Figure 7:**
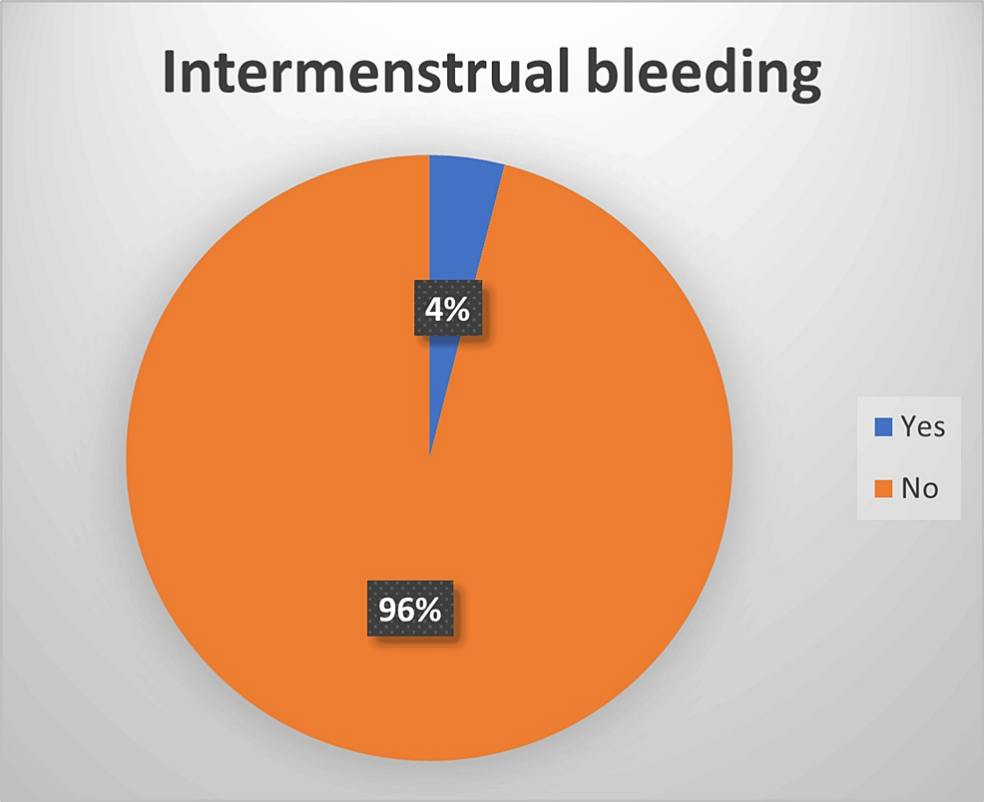
Intermenstrual bleeding

Only 4% of our participants reported new-onset intermenstrual bleeding (see Figure 7), which is in line with Chourasia et al.’s finding that women had less bleeding between periods during and after infection than before infection [2,3,12]. This is similar to a study by Al-Najjar et al., which showed that 18% of women had intermenstrual bleeding after infection [15].

Only 3% of our participants were postmenopausal, and only 1% of those reported postmenopausal bleeding. Although there has been research on COVID-19 vaccination and postmenopausal bleeding, we did not find any papers on the topic after COVID-19 infection.

Three studies on the relationship between COVID-19 infection and changes in the menstrual cycle were included in a systematic review by Lebar et al. showing that both menstrual volume and cycle length were affected by COVID-19 infection where the severity of infection had no bearing on the changes. This review, however, is not without limitations, as there is a paucity of research on this topic, and only three suitable articles were reviewed [16].

Although our study does not show significant menstrual pattern changes from COVID-19 infection, a systematic review by Rehan et al. revealed three articles evaluating the possible effects of COVID-19 infection on menstrual health and showed an escalation in symptoms of premenstrual syndrome, oligomenorrhea, and irregular cycles in women who suffered from COVID-19 infection [17].

A recent study by Li et al., published in March 2023, concluded that abnormal changes in menstruation also appeared in some women with COVID-19, including worsening premenstrual symptoms, prolonged menstrual cycles, and decreased menstrual volume; however, most menstrual changes recovered within a short period of time [18]. This indicates the transient nature of menstrual symptoms that occur postinfection. Menstrual changes were more common in patients with systemic complications arising from COVID-19 [19].

A study by Madaan et al. concluded that SARS-CoV-2 may infect the ovary, uterus, and vagina, leading to disturbances of female reproductive system function, such as infertility and various menstrual disorders [20].

A cross-sectional study by AlShrouf et al. on the effect of COVID-19 on the menstrual cycle concluded that infected women showed markedly increased menstrual aberrations. Furthermore, these women were comparably older in age, had had more intense symptoms, and were more likely to have had irregular menstruation during the pandemic [21].

In a review by Carp-Veliscu et al. on the effect of COVID-19 infection on female infertility, oligomenorrhea, menorrhagia, exacerbated premenstrual symptoms, missed periods, and decreased libido were among the findings with no statistical significance in menstrual volume changes between patients with mild and severe illness [22].

Stress is also an overlapping factor in causing menstrual irregularities in women with COVID-19 infection. A cross-sectional online survey study assessed the impact of COVID-19 infection and stress and showed that the degree of anxiety and stress as a result of the COVID-19 outbreak increased high enough to affect the characteristics of the menstrual cycle in the women surveyed [23]. Another study suggests that the COVID-19 pandemic may have directly contributed to menstrual cycle irregularities in women experiencing both moderate and high degrees of stress [24].

There is overlap between the menstrual changes experienced by women after the COVID-19 infection to those after the COVID-19 vaccination. These identical manifestations of the COVID-19 infection and COVID-19 vaccination have been highlighted by the studies done on the effect of COVID-19 vaccination on the menstrual cycle. It is worthwhile to note that, similar to the infection, the changes occurring after the vaccination are also transient.

A cross-sectional study surveyed the effect on menstrual cycle after both the infection and COVID-19 vaccination; it showed that 35.7% of those who had COVID-19 and 15.1% of those who had COVID-19 vaccine reported various menstrual irregularities [25].

A study by Tandon et al. showed that COVID-19 vaccinations have been associated with menstrual irregularities in females. It is imperative, however, to note that these changes were only transient. The study looked into five aspects of the female menstrual cycles: the regularity, length of the cycle and menstrual period, flow, and pain. The study observed a significant impact on the regularity, length of the menstrual cycle and pain during menstruation; however, all these changes were temporary [26].

Another study by Aljehani et al. observed a frequency of menstrual irregularities in Saudi women of child bearing age after COVID-19 vaccination, mostly affecting the cycle duration and dysmenorrhea [27].

Limitations

As our hospital is a women and maternity hospital, our study population included some women who were pregnant at the time of infection.

## Conclusions

The findings of this survey indicate that COVID-19 does not significantly affect the female reproductive system in terms of menstrual cycle changes. The majority of participants did not experience any new onsets of dysmenorrhea, changes in menstrual flow rate, amenorrhea, intermenstrual bleeding, or postmenopausal bleeding. However, further prospective studies are needed in this area.

## Appendices

Effect of COVID-19 in women: Questions related to COVID-19 symptoms and menstrual cycle

You are invited to participate in the study entitled “Effect of COVID-19 in women.” The purpose of this study is to evaluate the effect of COVID-19 on female reproductive system. Your participation will involve completing an anonymous questionnaire that will take about 10 to 15 minutes. Your participation in this survey study is voluntary. You may choose not to participate. If you decide to participate in this research survey, you may withdraw at any time. There are no anticipated risks to participating in this questionnaire and project. This study will not provide direct benefits to participants; however, participant will share information regarding COVID-19 and the menstruation cycle. The results and conclusion of this project will highlight relevant information regarding COVID-19 and women’s reproductive health. Your responses will be confidential, and we do not collect identifying information such as your name, email address, etc. For the sake of confidentiality, the completed questionnaire will be stored in a safe and secured folder.

*Required

Section I

1. Your age (in years)*

2. Postmenopausal:

- Yes

- No

3. Which of the following best describes you?*

-White

-Black or African American

-Asian

-Middle Eastern or North African

-Some other race, ethnicity or origin

4. Country of origin*

5. Date of COVID-19 infection (DD/MM/YYYY):*

6. Do you currently have a chronic condition?*

-Yes

-No

7. If you have chronic conditions, please indicate.

-Diabetes mellitus

-Cardiac disease

-Chronic hepatitis

-Pulmonary disease

-Dyslipidemia

-Stroke

-Anxiety/depression

-Cancer

-Obesity

8. Symptoms during the COVID-19 infection*

-No symptoms (asymptomatic)

-Fever

-Chills

-Headache

-Nasal congestion

-Sore throat

-Hoarseness (dysphonia)

-Dry cough

-Cough with sputum

-Muscle pain (myalgia)

-Weakness (fatigue)

-Chest tightness

-Shortness of breath (dyspnea)

-Abdominal pain

-Diarrhea

-Conjunctivitis

-Rashes on the skin

-Loss of taste and smell

9. During the COVID-19 infection, you were treated at*

-Home

-Home under doctor consultation

-Admitted to a hospital

10. If you were treated at home or home under doctor consultation, did you take medicines?

-Yes

-No

11. If you were admitted to a hospital, how many days you stayed in the hospital?

-Less than 5 days: Yes/No

-Between 5-10 days: Yes/No

12. If you were admitted to a hospital, you were given

-Oxygen treatment: Yes/No

-High flow nasal cannula: Yes/No

-Mechanical ventilation: Yes/No

13. If you were admitted to a hospital, did you receive medication specific for COVID-19?

-Yes

-No

14. Effect on menstrual cycle?

1. New-onset dysmenorrhea (painful periods)

-Yes

-No

2. Menstrual flow

-Scanty: Yes/No

-Heavy: Yes/No

3. Amenorrhea

-Yes

-No

4. Intermenstrual bleeding

-Yes

-No

5. Postmenopausal bleeding

-Yes

-No

-N/A
